# Molecular cloning and characterization of five *SmGRAS* genes associated with tanshinone biosynthesis in *Salvia miltiorrhiza* hairy roots

**DOI:** 10.1371/journal.pone.0185322

**Published:** 2017-09-27

**Authors:** Zhenqing Bai, Pengguo Xia, Ruilin Wang, Jie Jiao, Mei Ru, Jingling Liu, Zongsuo Liang

**Affiliations:** 1 College of Life Science, Northwest A&F University, Yangling, China; 2 College of Life Science, Zhejiang Sci-Tech University, Hangzhou, China; Clemson University, UNITED STATES

## Abstract

The gibberellin-responsive element binding factor (GRAS) family of proteins plays an important role in the transcriptional regulation of plant development and hormone signaling. To date, there are no reports on GRAS family proteins expressed in *Salvia miltiorrhiza*. In this study, 28 ESTs that contained the *GRAS* domain were identified from a *S*. *miltiorrhiza* cDNA library. Of these, full-length sequences of five genes were cloned and sequence analysis indicated that all five proteins contain only one GRAS domain and therefore, belong to the GRAS family. The five genes were designated *S*. *miltiorrhiza GRAS1-5* (*SmGRAS1-5*), which belong to groups I (*SmGRAS2* and *SmGRAS4*), II (*SmGRAS3*), III (*SmGRAS1*), and VIII (*SmGRAS5*) respectively. Additionally, *SmGRAS1-5* have different expression patterns in the reed head, stems, leaves, flowers, and roots of *S*. *miltiorrhiza*. In this study, the expression of *SmGRAS1-5* was sensitive to Gibberellin (GA) stress and that of *SmGRAS1*, *SmGRAS4* and *SmGRAS5* was sensitive to Ethephon (Eth) stress respectively. Moreover, *S*. *miltiorrhiza* copalyl diphosphate synthases 1 (*SmCPS1*) and *S*. *miltiorrhiza* kaurene synthase like 1 (*SmKSL1*), which are two key enzymes gene in the diterpenoid biosynthesis pathway, were also response to GA and Eth stress. In addition, Dihydrotanshinone (DT-I) and Tanshinone I (T-I) content were enhanced by GA and Eth stress, Tanshinone IIA (T-IIA) content was increased by GA stress, and the accumulation of Cryptotanshinone (CT) was insensitive to both GA and Eth stress. Together, these results provide insights into functional conservation and diversification of SmGRASs and are useful information for further elucidating SmGRAS functions.

## Introduction

Plant growth and yield is strongly influenced by hormones such as GA, Jasmonate (JA), Salicylic acid (SA) and Eth [1]. Plants respond and adapt to these signals through an array of biochemical and physiological changes, including the regulation of adaptation processes by stress responsive gene expression [1]. Transcription factors play a central regulatory role in gene expression, and the GRAS proteins have important functions in the transcriptional regulation of a variety of biological processes such as plant development, shoot apical meristem maintenance [2, 3], and response to abiotic stresses and nodulation signaling [4]. The GRAS proteins contain conserved residues at their C-terminal and a variable N-terminal domain [5]. Typically, GRAS proteins exhibit motifs with sequence homology to each other in their C-terminus, including a leucine heptad repeat I (LHR I), the VHIID motif, the leucine heptad repeat II (LHR II), the PFYRE motif, and the SAW motif [2, 6, 7]. Among them, the VHIID motif, the name of which is derived from the most prominent constituent amino acid residues, is present in all members of the GRAS family, and only the histidine and the aspartic acid are absolutely conserved [2]. The LHR I and LHR II have approximately 100 amino acid residues that are characterized by largely leucines residues, and could be important for protein–protein interactions [2]. ([RK]-x (2,3)-[DE]-x (2,3)-Y) is overlapping with the tyrosine in the PFYRE motif, and functions as a phosphorylation site [8]. The SAW motif contains three pairs of conserved residues: R (x)4 E, W (x)7 G, W (x)10 W, and functions as a regulatiory domain [2]. GRAS proteins contain eight subfamilies: *DELLA*, *HAIRY MERISTEM (HAM)*, *SCARECROW-like (SCL) PHYTOCHROME A SIGNAL TRANSDUCTION1 (PAT1)*, *LATERAL SUPPRESSOR (LS)*, *SCARECROW (SCR)*, *SHORTROOT (SHR)*, *SCARECROW LIKE3 (SCL3)* [2]. The *SCR* and *SHR* genes are involved in radial organization of the root through the formation of a *SCR/SHR* complex [9]. *SCL3* acts as an integrator downstream of the GA/DELLA and *SCR/SHR* pathways, mediating GA-promoted cell elongation during root development [10, 11]. The *PAT1*, *SCL5*, *SCL13* and *SCL21* genes are highly homologous in *Arabidopsis thaliana* and are involved in light signaling pathways. Meanwhile, *PAT1*, *SCL5*, *SCL21* are positive regulators of phytochrome-A signal transduction, while *SCL13* mainly participates in phytochrome-B signal transduction [12–14]. *AtRGL2* is involved in seed germination and is up-regulated during *A*. *thaliana* seed imbibition [15].

There are many reports on *GRAS* in response to abiotic and biotic stress. For example, *SlGRAS* (*Solanum lycopersicum*) is expressed in response to the stress induced by ethephon (Eth), gibberellic acid (GA), indole acetic acid (IAA), salicylic acid (SA), and abiotic factors in tomato [16]. The stress-inducible *GRAS* gene *VaPAT1* (*Vitis amurensis*) enhances cold, drought, and salt tolerance in transgenic *A*. *thaliana* by modulating the expression of a series of stress-related genes [17]. In response to low phosphate conditions, *LiGRAS* (*Lotus japonicas*) can display a phylogenetic pattern characteristic of symbiotic genes [18].

Tanshinone, obtained from the roots of *S*. *miltiorrhiza*, is a major ingredient in traditional Chinese medicines.[19]. Tanshinone is mainly synthesized via the 2-C-Methyl-D-erythritol 1, 2-cyclophosphate 4-phosphate (MEP) pathway in the plastids, and to some extent also via the mevalonic acid (MVA) pathway in the cytoplasm [20]. Have many reports showed that these pathways were in response to GA and Eth [21–25]. Meanwhile, many reports have shown that *GRAS* genes have an affect on MEP and MVA pathways. For example, *AtSCL3* influences GA signaling and homeostasis [10, 11]. Furthermore, *AtPAT1*, *AtSCL13*, and *AtSCL6* are involved in phytochrome A, phytochrome B, and GA signaling, respectively [7, 14]. To better understand the function of *SmGRAS* proteins in tanshinone metabolism, we searched and amplified *SmGRAS* genes from the *S*. *miltiorrhiza* transcriptome database. This research focused on the prediction of the functions of these *SmGRAS* genes and their response to GA and Eth treatments. Additionally, downstream of the MEP and MVA pathways that are involved in the tanshinone synthesis pathway in *S*. *miltiorrhiza*, SmCPS1 catalyzes (E,E,E)-geranyl- -geranyl diphosphate (GGPP) to produce copalyl diphosphate (CPP), and then SmKSL1 catalyzes CPP to form miltiradiene, which is a precursor for tanshinone biosynthesis [20]. SmCPS1 and SmKSL1 are key enzymes that function in the last step of tanshinone biosynthesis [20, 26, 27]; we also observed gene expression levels of *SmCPS1* and *SmKSL1* under GA and Eth stress. The changes in tanshinone content were detected under the GA and Eth stress. Finally, *SmGRAS* response to GA and Eth signaling and its involvement in tanshinone biosynthesis were analyzed.

## Materials and methods

### Plant materials and growth conditions

The *S*. *miltiorrhiza* sterile plantlets seedlings, which preserved in our lab, were cultured on an MS medium (pH 5.8) that contained 7% agarose. The hairy roots of *S*. *miltiorrhiza* were derived from these plantlets, infected with *Agrobacterium rhizogenes* (ATCC15834 strain), and sub-cultured every 30 days.

For GA and Eth treatments, 18-day-old *S*. *miltiorrhiza* hairy roots were incubated in 6,7-V liquid medium in a shaker (25°C, 120 rpm) with 50 mg/L GA and 200 μg/L Eth (Sigma-C0143) for 0 h, 1 h, 2 h, 4 h, 8 h, 12 h, 24 h, 72 h, and 8 d. Treated samples were harvested separately for each incubation period. *S*. *miltiorrhiza* hairy roots grown in liquid 6,7-V medium without GA or Eth were used as controls. Here we set three biological replicates, and each replicate contained five hairy roots.

To analyze tissue-specific *SmGRAS* gene expression, leaves, roots, stems, flowers, and reed heads were collected when the *S*. *miltiorrhiza* seedlings were at the flowering stage.

### Gene clone and bioinformatic analyses of *S*. *miltiorrhiza GRAS* genes

The full-length ORF sequences of five *S*. *miltiorrhiza GRAS (SmGRAS)* were extracted from the Danshen transcriptional Resource Database (DsTRD: http://bi.sky.zstu.edu.cn/DsTRD/home.php). The full-length *SmGRAS* coding sequences (CDSs) were amplified by PCR using the primers listed in supplementary S1 Table. PCR products were gel-purified, cloned, and sequenced. An e-value cut-off of 1e-10 was applied for homologue recognition. The retrieved sequences were used for gene model prediction on the GENSCAN web server (http://genes.mit.edu/GENSCAN.html). The theoretical isoelectric point (pI) and molecular weight (Mw) were predicted using the pI/Mw tool on the ExPASy server (http://web.expasy.org/computepi/). Phylogenetic trees were constructed using the maximum likelihood method with MEGA6.0. The resulting tree topology was reassembled by blasting the *A*. *thaliana* database [7]. Conserved motifs were investigated using multiple alignment analyses with MEME version 3.0. The prediction of nucleus positioning signal peptides was performed with the online web tool, http://nls-mapper.iab.keio.ac.jp/cgi-bin/NLSMapper–form.cgi.

### RNA isolation and quantitative RT-PCR analysis of *SmGRAS* genes

Total RNAs from *S*. *miltiorrhiza* Bunge roots were extracted using the TIANGEN reagent according to the manufacturer’s instructions (TIANGEN, China). The DNase-treated RNA was reverse transcribed using SuperScript reverse transcriptase (TaKaRa, China) according to the manufacturer’s instructions. Real-time quantitative PCR was performed on an optical 96-well plate with an ABI PRISM 7500 real-time PCR system (Applied Biosystems) using SYBR Premix ExTaq (TaKaRa, China). The PCR method was programmed as follows: 95°C for 30 s, followed by 40 cycles at 95°C for 5 s, and 60°C for 30 s. The *SmActin* gene was used as the endogenous control. The quantitative RT-PCR primer is shown in supplementary S2 Table. These experiments were repeated three times.

### HPLC analysis

The high performance liquid chromatography (HPLC) method involved a reference and was performed according to the method established in our laboratory [28]. We added 8 ml 70% methanol to 0.04 g dry sample, allowed the sample to incubate overnight at room temperature, and then it was sonicated for 45 min, followed by centrifugation at 8,000 rpm for 10 min. The sample was then filtered through a 0.45-μm filter. HPLC analysis was performed using a Waters (Milford, MA, USA) system with a binary pump and photodiode array detector. A Sun Fire C18 column (250 × 4.6 mm, 5 μm; Waters) was used. The flow rate was 1 ml/min, the column temperature was 30°C, and the sample volume used was 10 μl.

### Data statistics and analysis

Experiments were performed with three biological and technical replicates, respectively. The relative gene quantification method (delta-delta Ct) was used to evaluate quantitative variation in gene expression. The ANOVA statistical analysis of HPLC data, and 2-ΔΔCq was used to assess the qRT-PCR results. ANOVA (analysis of variance) followed by the least significant difference (LSD) test were calculated in SPSS (Version 19.0, IBM, USA) and considered statistically different at *p*<0.05 or *p*<0.01. Error bars indicate the standard deviation obtained from three different experiments. The figures were drawn using Origin 9.0 software.

## Results

### Sequence features of *SmGRASs* in *S*. *miltiorrhiza*

In this study, 28 ESTs whose amino acid sequences contained a GRAS domain were identified from a *S*. *miltiorrhiza* cDNA library. Of these, full-length coding sequences (CDS) of five genes were cloned and our analysis indicated that all five proteins contained only one conserved GRAS domain, suggesting that they belong to the GRAS protein family. We designated the genes as gibberellin-responsive element binding factor 1–5 (*SmGRAS1–5*). The *SmGRAS1–5* gene IDs are KY435886, KY435887, KY435887, KY435888, KY435889, and KY435890, respectively. The ORFs of the five genes did not contain introns and encoded 489, 459, 748, 526, and 335 amino acids, respectively. Furthermore, nucleus location signals (NLS) were identified in the five *SmGRAS* genes. These five SmGRAS proteins belong to groups I (SmGRAS2 and SmGRAS4), II (SmGRAS3), III (SmGRAS1), and VIII.(SmGRAS5) These groups may play a role in phytochrome A and B, light, and GA signal transduction pathways during plant growth and development [2, 3]. The SmGRAS proteins were also clustered into the SHR and SCR-like (SCL) subfamily (S3 Table).

### Phylogenetic analysis and architecture of conserved motifs in SmGRAS proteins

**Fig 1:**
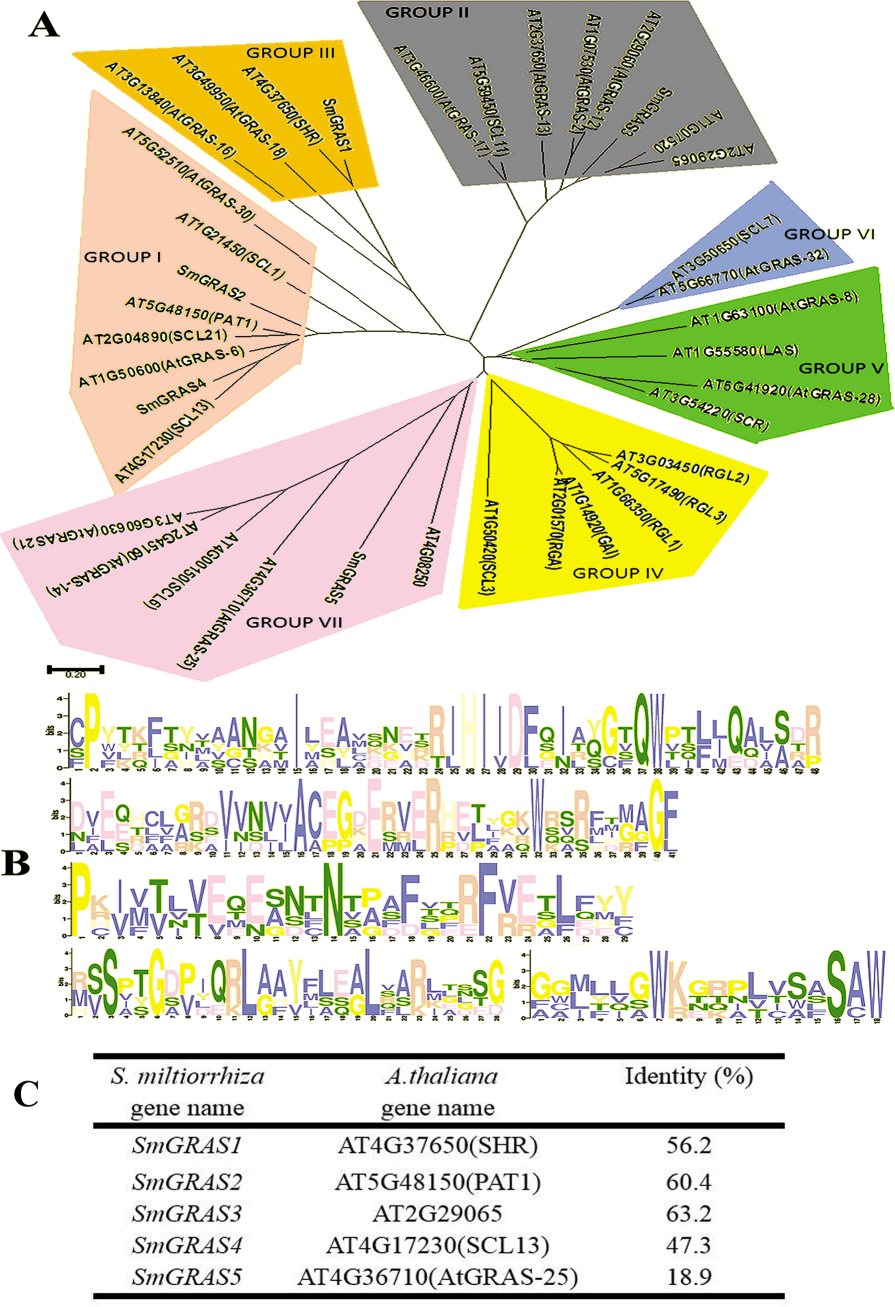
Phylogenetic tree and architecture of conserved protein motifs of SmGRASs. (A), The maximum-likelihood tree of SmGRAS proteins compared to AtGRAS proteins. Bootstrap values were shown at each node. (B), The architecture of five SmGRAS proteins shows their conserved protein motifs. (C). The amino acid sequence identity blast between SmGRAS and AtGRAS.

The AtGRAS protein sequence, which was used for the *SmGRAS* phylogenetic tree construction, was identified from *A*. *thaliana* protein databases. The phylogenetic tree showed that the five SmGRAS proteins developed from changes in gene structure over the course of *S*. *miltiorrhiza* evolution. The SmGRAS1 clustered into group III, SmGRAS2 and SmGRAS4 clustered into group I, SmGRAS3 clustered into group II and SmGRAS5 clustered into groups VII (Fig 1A). Over the course of the evolution of this gene, it most likely expanded, leading to these structural and functional changes. Furthermore, the five conserved domains of SmGRAS proteins were identified and marked (Fig 1B). By protein sequence alignment, we find that 56.2% amino acids are identical between SmGRAS1 and AtSHR, SmGRAS2 has 60.4% identity to AtPAT1, and SmGRAS3 has 63.2% identity to At2G29065, while the identity between SmGRAS4, SmGRAS5 and any AtGRAS proteins is no more than 50% (Fig 1C). Besides, SmGRAS1 shared similar protein sequence-structure with AtSHR1, which is involved in shoot-root growth regulation [7]. SmGRAS4 is most close to AtSCL13 whose function is still unknown. None of the AtGRAS member is located in the same clade with SmGRAS2, SmGRAS3 or SmGRAS5. However, SmGRAS2 was similarity with AtPAT1 (Fig 1A and 1C), whose functions in the phytochrome A signal transduction [12].

### Tissue specific expression levels of *SmGRAS* genes

**Fig 2:**
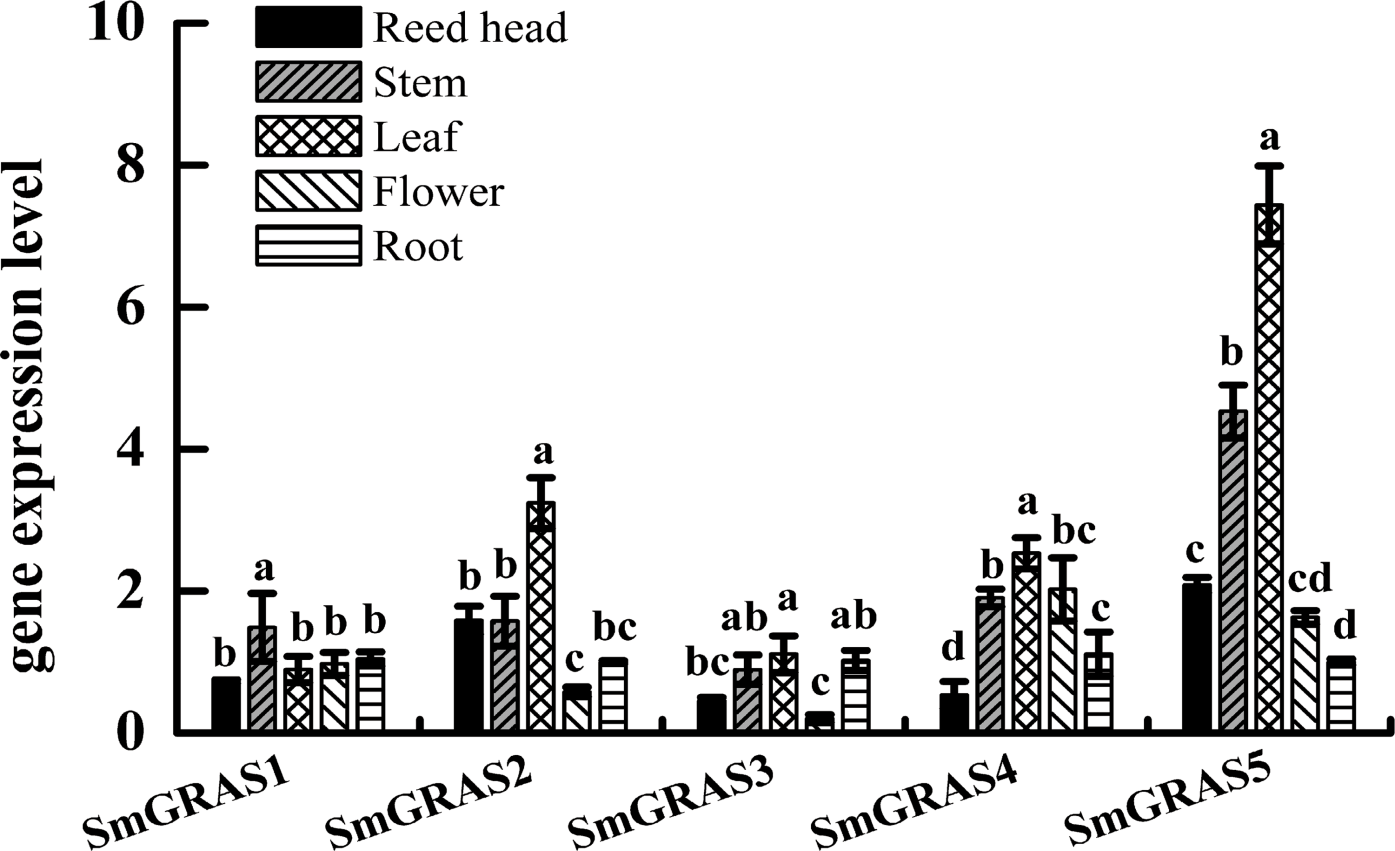
The expression pattern of the five *SmGRASs* gene in different tissues (root, stem, leaf, flower, and reed head). reed head: Instead of the joint between root and stem. Data are shown as means ±SD (n = 3) and the lowercase letters indicate statistical significance at *p* < 0.05.

In *S*. *miltiorrhiza*, the five GRAS genes identified had tissue-specific expression profiles that could inform their differentiated roles in tissue or plant development. The tissue expression profiles are presented in Fig 2. *SmGRAS1* was expressed in reed heads, roots, stems, leaves, and flowers; *SmGRAS2* was mainly expressed in the reed head, leaf, and stem; *SmGRAS3* had higher expression level in the root, leaf, and stem; *SmGRAS4* was expressed at a higher level in the leaf, stem, and flower; and *SmGRAS5* was expressed in the leaf and stem. In stem and leaf, the expression level of *SmGRAS5* was highest in five *SmGRAS* genes. In flower, the expression level of *SmGRAS4* higher than others. In the reed head, *SmGRAS2* and *SmGRAS5* have the higher expression level than others. In the root, the *SmGRAS1~5* have not differences in their expression level. Therefore, these characteristics of *SmGRAS1-5* may be due to the changes in the gene structure of *SmGRAS* genes, and their close evolutionary relationships with *AtSCL* and *AtSHR* (Fig 1A), which are involved in phytochrome A and B signaling, GA signaling, and regulating shoot-root development [2, 7, 9].

### The gene expression level of *SmGRAS* and *SmCPS1* and *SmKSL1* genes in response to GA treatment

**Fig 3:**
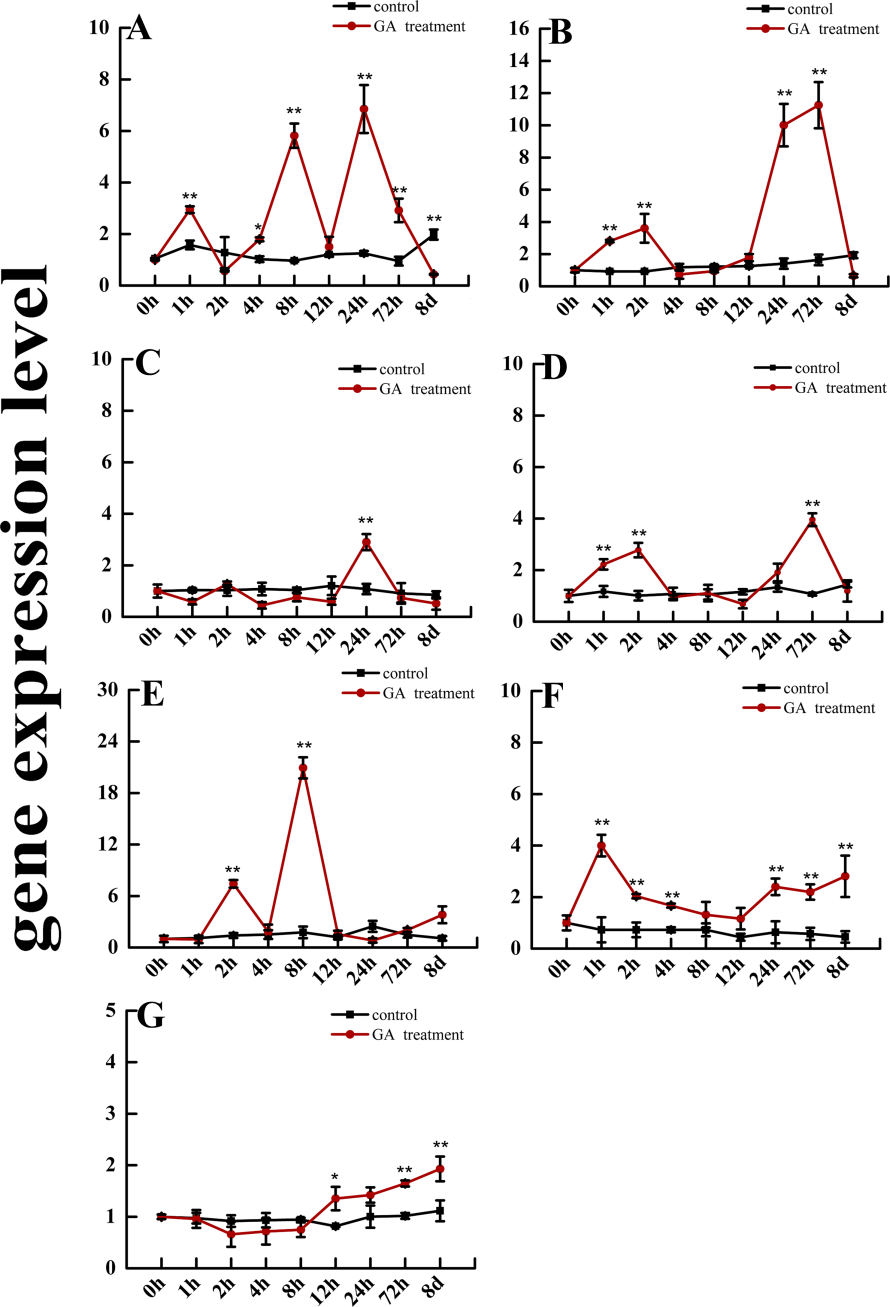
The gene expression level of *SmGRAS1-5* (A-E), *SmCPS1*(F), and *SmKSL1*(G) genes in response to GA treatment. Data are shown as means ±SD (n = 3); the single asterisk indicates significance at *p* < 0.05 and the double asterisk indicates significance at *p* < 0.01.

*GRAS* gene products play an important role in terpenoid biosynthesis [7, 10, 11, 13]; therefore, *SmGRAS1*-5 may also have an evolutionarily conserved role in regulating terpenoid biosynthesis. Among the five identified *SmGRAS* genes, the expression levels of *SmGRAS1* increased in the early stages of GA treatment, except at 2 h and 12 h (*p*<0.05 or *p*<0.01), and then decreased significantly after 8 days (Fig 3A). The gene expression levels of *SmGRAS2* and SmG*RAS4* were the highest at 2 h and 72 h of GA treatment, respectively (*p*<0.01). The gene expression *SmGRAS2* at 1 h and 24 h and *SmGRAS4* at 1 h significantly were upregulated by the GA treatment (*p*<0.01). *SmGRAS3* expression decreased in the initial stage of treatment and then increased until 24 h after treatment (*p*<0.01). The gene expression level changes showed that *SmGRAS5* was sensitive to exogenous GA stress, especially after 2 h and 8 h (*p*<0.01) of GA treatment. In addition, the gene expression levels of *SmCPS1 and SmKSL1*, which encode the key enzymes in tanshinone biosynthesis, were measured. The expression of *SmCPS1* was significantly up-regulated after 1 h, but not at 8 h and 12 h of GA treatment (*p*<0.01) (Fig 3F); however, the expression levels of *SmKSL1* decreased slightly in the early stages of GA treatment, and then continued to increase significantly after 12 h till the 24 h time point (*p*<0.05 or *p*<0.01). Thus, the expression of the *SmGRAS1-5* and *SmCPS1* and *SmKSL1* genes were all responsive to GA stress and exhibited spatiotemporal relationship to each other.

### The gene expression level of *SmGRAS*, *SmCPS1*, and *SmKSL1* genes in response to Eth treatment

**Fig 4:**
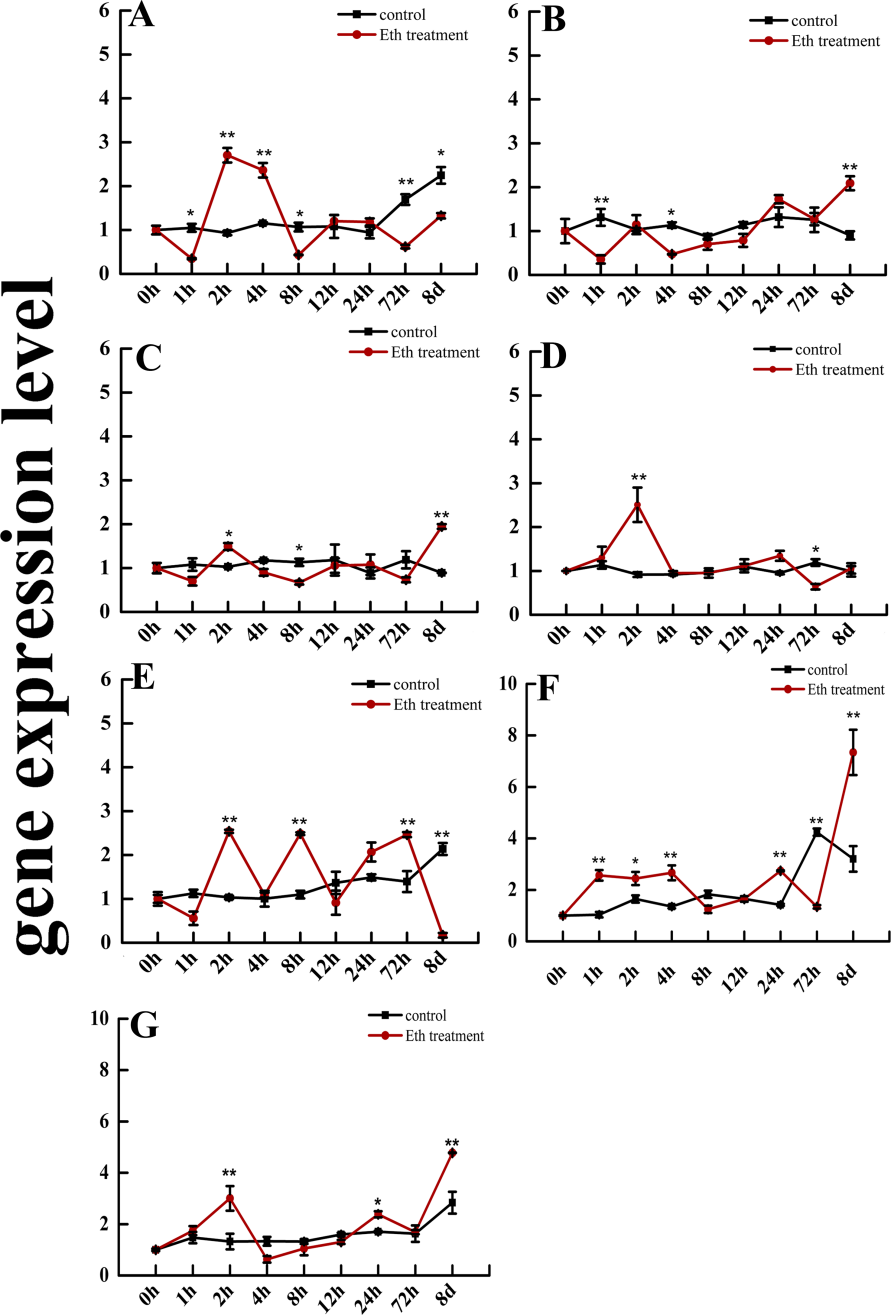
The gene expression level of *SmGRAS1-5* (A-E), *SmCPS1* (F), and *SmKSL1* (G) in response to Eth treatment. Data are shown as means ±SD (n = 3); the single asterisk indicates significance at *p* < 0.05 and the double asterisk indicates significance at *p* < 0.01.

In order to determine if the *SmGRAS* genes respond to Eth signaling, we measured gene expression levels of *SmGRAS1-5* during Eth treatment. The gene expression of *SmGRAS1* decreased after 1 h, 8 h, and 72 h (*p*<0.01), and significantly increased at 2 h and 4 h (*p*<0.01) of treatment. However, *SmGRAS2* decreased at 1 h, 4 h, 8 h, and 12 h, but then increased after 24 h of Eth treatment. The lowest expression level of *SmGRAS2* was measured at 1 h of treatment and was the highest after 8 days of Eth exposure (*p*<0.01). *SmGRAS3* increased at 2 h and 8 days (*p*<0.01), but the expression decreased at the other treatment times. *SmGRAS4* was significantly upregulated by Eth treatment after 2 h (*p*<0.01) and *SmGRAS5* showed a wave-like response to Eth stress at 2 h, 8 h, and 72 h (*p*<0.01). In addition, the gene expression level of *SmCPS1* significantly increased at the 1 h, 2 h, 4 h, and 8 day time points under Eth stress (Fig 4F); but its expression declined at 8 h, 12 h, and 72 h after treatment (*p*<0.01). Like the expression pattern of *SmCPS1*, *SmKSL1* was significantly induced by Eth stress at 2 h, 24 h, and 8 days, respectively (Fig 4G). Thus, the *SmGRAS1-5*, *SmCPS1*, and *SmKSL1* genes respond to Eth stress.

### The accumulation of DT-I, T-I, and T-IIA but not CT are sensitive to exogenous GA and Eth stress in *S*. *miltiorrhiza* hairy roots

**Fig 5:**
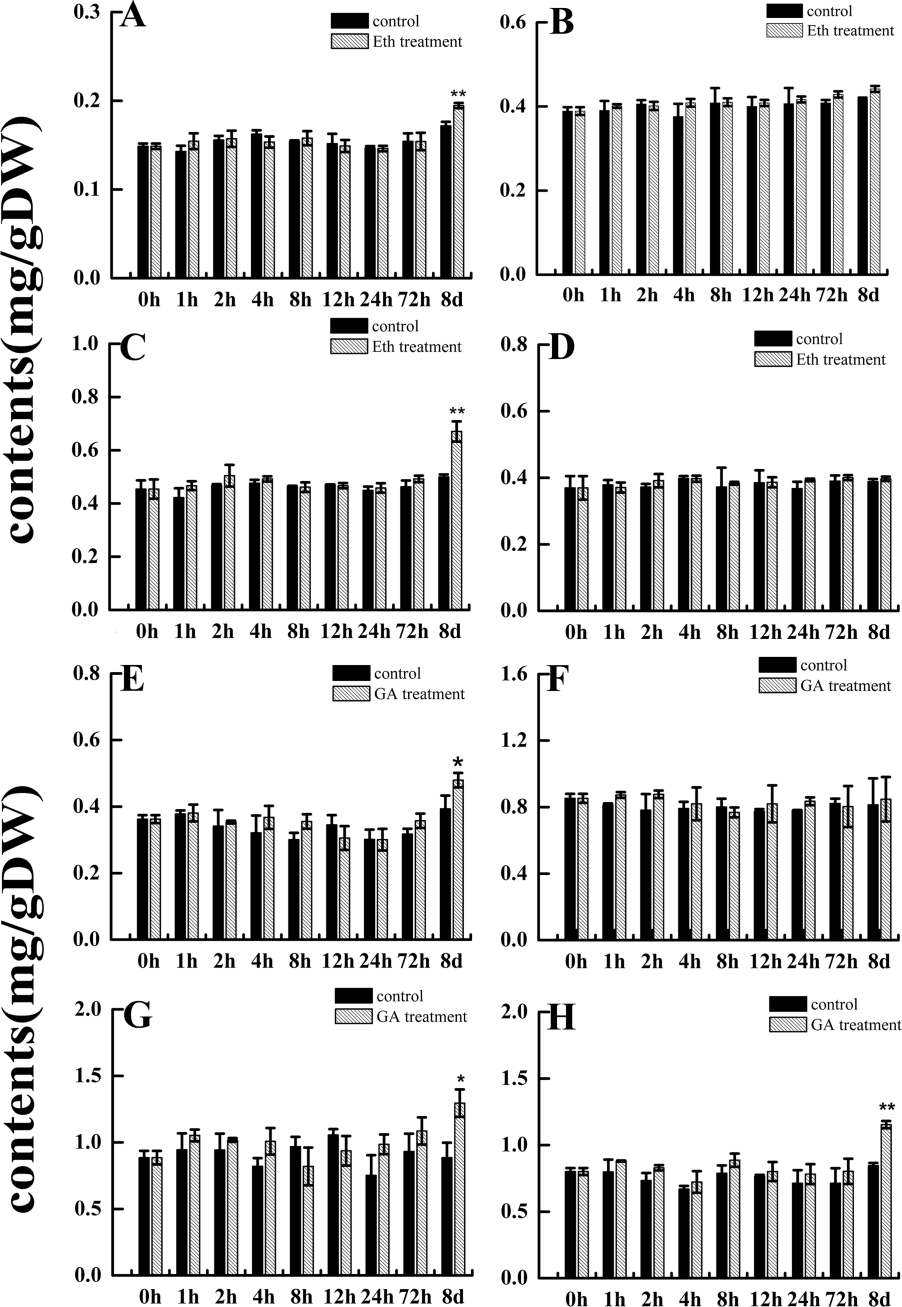
The tanshinone content of *S*. *miltiorrhiza* hairy roots under GA and Eth treatments. A-D: DT-I, CT, T-I, and T-IIA content changes over time with exogenous Eth treatment, respectively. E-H: DT-I, CT, T-I, and T-IIA content changes over time with exogenous GA treatment, respectively. Data are shown as means ±SD (n = 3); the single asterisk indicates significance at *p* < 0.05 and the double asterisk indicates significance at *p* < 0.01.

The secondary metabolism of *S*. *miltiorrhiza* can be induced by exogenous biotic and abiotic factors. Tanshinone mainly contains the dihydrotanshinone (DT-I), cryptotanshinone (CT), tanshinone I (T-I), and tanshinone IIA (T-IIA) compounds in *S*. *miltiorrhiza*. Our study used exogenous GA and Eth to treat *S*. *miltiorrhiza* hairy roots. The results showed an accumulation of DT-I and T-I induced by Eth (*p*< 0.01) on the eighth day of treatment (Fig 5A and 5C). However, CT and T-IIA did not accumulate with exogenous Eth treatment. During GA treatment, the accumulation of DT-I, T-I, and T-IIA significantly increased compared to the control after 72 h (Fig 5E and 5G), especially after eight days (*p*< 0.05 or *p*< 0.01). Conversely, the accumulation of CT was insensitive to exogenous GA signaling. Our results showed that the accumulation of tanshinone compounds was not significantly different from the control at the preliminary stages of exogenous GA and Eth stress, but did differ significantly in their accumulation after eight days of treatment with either exogenous compound. These results suggest that the accumulation of tanshinone compounds is sustainable under exogenous GA and Eth stress.

## Discussion

Many TFs take part in plant secondary metabolite regulation [29], and the induced accumulation of secondary metabolites is usually strictly regulated by TFs [30]. Many TFs that participate in the transcription regulation such as *MYB*, *bHLH*, *AP2/ERF*, *WRKY*, *SPL* and *NAC* have been verified [30–32]. In addition, *GRAS* family genes, which specifically respond to GA signaling, play important roles in the regulation of diterpenoid biosynthesis [7, 10, 11, 14]. The *GRAS* family is found in several plant species, including *Populus trichocarpa*, *A*. *thaliana*, *Oryza sativa*, *Brassica pekinensis*, *Prunus mume*, and *Pinus radiata* [33–36]; however, only part of the *GRAS* protein family is functionally characterized in *Zea mays*, Petunia hybrids, *Medicago truncatula*, and *Lilium longiflorum* [37–40]. For example, *AtSCL14* interacts with a TGA product, and this complex seems to be involved in the activation of a general broad-spectrum detoxification network upon challenge with xenobiotics like auxin and SA [41].

The *GRAS* family is one type of plant specific transcription regulatory factors. The originally discovered members of the family are *GAI*, *RGA* and *SCR*, which named by the characteristics of the alphabet [2]. The GRAS family proteins have different roles in metabolic pathways throughout plant development. These members include *SHR*, *SCR*, *LS*, *HAM*, *PAT1*, and *DELLA*, which are involved in biological metabolism processes of root radial growth, the growth of axillary buds, meristem maintenance, phytochrome signaling pathways, and GA signaling pathways [7]. By analyzing sequence identity, we find that the amino acid of SmGRAS1, SmGRAS2 and SmGRAS3 have about 60% amino acid identity to AtSHR, AtPAT1 and At2G29065 respectively. However, only low amino acid identity existed between SmGRAS4, SmGRAS5 and AtGRAS (Fig 1C). In *A*. *thaliana*, the group AtSHR, AtPAT1, AtSCL1, AtSCL11 and AtSCL6 are related to phytochrome A, phytochrome B, shoot-stem development, abiotic stress response and GA signaling, respectively, however, AtSCL13 function is unknown [7, 42]. Thus, SmGRAS proteins might fill a role in phytochrome A, phytochrome B, shoot-stem development, abiotic stress response and GA signaling respectively in *S*. *miltiorrhiza*. In this study, the *SmGRAS1-5* have different expression patterns in the reed head, stem, leaf, flower, and root. Indeed, *SmGRAS2*, *SmGRAS3*, *SmGRAS4*, and *SmGRAS5* had the highest expression levels in leaves than in any other tissues. This was consistent with the phylogenetic results that arranged these genes closed to the SCL subgroup, suggested they might have a relationship with phytochrome A, phytochrome B and GA signaling [7]. However, *SmGRAS1* was expressed in all tested tissues and was closely aligned with AtSHR, indicating that it might be involved in shoot-stem development [7]. In addition, *SmGRAS4* was highly expressed in the flower, which further implies its role in phytochrome A and B function.

Many *GRAS* family members are important in responding to biotic and abiotic stresses. Earlier reports show that GRAS family members were expressed in response to low temperature, drought, infiltration, and hormones in *A*. *thaliana*, rice, bergamot, *P*. *euphratica*, and tobacco [43]. Thus, the GRAS family proteins have complex and diverse functions in plants. Now the mechanism of some members (e.g., DELLA) is relatively clear, but the mechanism for most GRAS protein members is not fully elucidated. DELLA proteins are key negative regulators in GA signaling, but also up-regulate gene expression of positive regulators in GA signaling, such as GA 20-oxidase, GA receptor, and the transcriptional regulator *SCARECROW-LIKE3 (SCL3)*, which enables the regulation of GA feedback [44]. In addition, GRAS family members are involved in plant stress responses, such as the tobacco *GRAS* gene (*NtGRAS1*) that is significantly up-regulated under H_2_O_2_ and salicylic acid stress [45]. In our study, *SmGRAS1-5* gene expression was sensitive to exogenous GA and Eth stress (Fig 3 and Fig 4). Specifically, *SmGRAS1*, *SmGRAS2*, and *SmGRAS5* were significantly sensitive to exogenous GA signaling (Fig 3A, 3B and 3E). Furthermore, *SmGRAS1*, *SmGRAS4*, and *SmGRAS5* were sensitive to exogenous Eth signaling (Fig 4A, 4D and 4E). In addition, under Eth treatment, *SmGRAS1* and *SmGRAS2* expression was significantly repressed at some time points and significantly induced at other time points. The expression level of *SmGRAS1* was also significantly decreased eight days after GA treatment (Fig 3A). These results are evidence of the feedback adjustment and adaption process on gene expression during exogenous GA and Eth stress [44]. Overall, *SmGRAS1-5* were responsive to exogenous GA and Eth signaling. The DELLA proteins play a key role in the crosstalk between GA and Eth signaling [46]; similarly, *SmGRAS1* and *SmGRAS5* expression is sensitive to GA and Eth signaling and this provides additional insight to GRAS protein function in the crosstalk between GA and Eth signaling.

Tanshinone is a secondary metabolite product of *S*. *miltiorrhiza* and the improvement of its production is an urgent solved problem to meet the needs of the market. In plants, genetic engineering is an effective strategy used to improve secondary metabolite production and there are some successful cases of genetic manipulation of natural plant activities, such as the overexpression of *S*. *miltiorrhiza* Geranylgeranyl pyrophosphate (*SmGGPPS*) and *S*. *miltiorrhiza* 1-deoxy-d-xylulose- 5-phosphate synthase (*SmDXSII)*, and the co-expression of *S*. *miltiorrhiza* hydroxy-3methylglutary CoA reductase (*SmHMGR*) and *SmGGPPS*, which produces higher levels of tanshinone in *S*. *miltiorrhiza* hairy roots [47–49]. Co-overexpression of geraniol-10-hydroxylase and strictosidine synthase can improve camptothecin accumulation in *Ophiorrhiza pumila* [50]. In addition, over-expressing tropinone reductase I and hyoscyamine-6b-hydroxylase enhanced the production of tropane alkaloids in transgenic *Anisodus acutangulus* hairy roots [51]. The research has developed transcription factors that are more effective on secondary metabolic regulation [29, 30]. The *SmCPS1* and *SmKSL1* are genes that encode key enzymes for diterpene biosynthesis [20, 26, 27]. Moreover, *SmGRAS1-5*, *SmCPS1*, and *SmKSL1* genes respond to GA and Eth stress. Combined with previous results, the stress-inducible expression patterns of the *SmGRAS* genes suggest that they might have a relationship with the gene expression regulation of *SmCPS1* and *SmKSL1*. As both tanshinone and GA are diterpenoids, their biosynthesis pathways are similar. These results implicate that *SmGRASs* are very likely to be transcriptional regulation activators of *SmCPS1* and *SmKSL1*. Moreover, GRAS protein function may be involved in diterpenoid biosynthesis; for example, *Phyllostachys edulis SCL3* (*PeSCL3*) may influence root development by regulating GA biosynthesis [52]. DELLA proteins play a key role in the crosstalk between GA and Eth signaling [46]. Thus, our results implicate increased *SmGRAS1-5* expression correlates with tanshinone biosynthesis increase under GA and Eth stress.

Reports on exogenous treatment with plant hormones or yeast components can elicit enhanced tanshinone accumulation in plant cell or tissue culture. For example, treatment with exogenous methyl jasmonate (MJ), salicylic acid (SA), Ag^+^, and yeast elicitors (YE) increased accumulation of tanshinone in *S*. *miltiorrhiza* hairy roots and transgenic *S*. *miltiorrhiza* hairy root lines [53–55]. Similar to these reports, our results show that the accumulation of DT-I and T-I was significantly induced by GA and Eth treatment after eight days, and the accumulation content of T-IIA was induced by GA treatment. However, the accumulation of CT content was insensitive to exogenous GA and Eth stress, and T-IIA was insensitive to Eth stress. As the tanshinone content production is a gradual accumulation process, the tanshinone content levels did not show significant change in the early time points after GA and Eth treatment. It was only after longer treatment periods that the tanshinone content accumulation was observed; suggesting that constant GA and Eth signaling stimulated this accumulation (Fig 5). Thus, these results support *SmGRAS1-5* participation in the regulation of tanshinone biosynthesis, which is mediated by GA and Eth signaling.

We amplified five *SmGRAS* genes from of *S*. *miltiorrhiza* and named them *SmGRAS1-5*, respectively. *SmGRAS1* was expressed in all tissues and has a close phylogenetic relationship with *AtSHR*. *SmGRAS2-5* were expressed at the highest levels in leaves, and share a close relationship with *AtPAT* and *AtSCL*. Our research shows that *SmGRAS1-5* expression is sensitive to exogenous GA, and *SmGRAS1*, *SmGRAS4*, and *SmGRAS5* is sensitive to exogenous Eth stress, while the *SmGRAS2* and *SmGRAS3* are either up-regulated or down-regulated at different time-points of Eth treatment. Interestingly, the gene expression of *SmCPS1* and *SmKSL1* increased with exogenous GA and Eth stress, as did the accumulation of DT-I and T-I content, while T-IIA content was induced only by GA stress after eight days. Overall, we show that *SmGRAS1-5* might participate in the regulation of tanshinone biosynthesis and are mediated by GA or Eth signaling. Thus, this study provides a resource for the selection of candidate genes for the further characterization of *S*. *miltiorrhiza* and enhancement of tanshinone production through the engineering of secondary metabolic pathways that could positively manipulate the production of target molecules in this “heal-all” medicinal plant.

## Supporting information

S1 Table The clone primers of *SmGRAS1~5*. (DOCX)

S2 Table The qRT-PCR primers of *SmGRAS 1~5*. (DOCX)

S3 Table Sequence features analysis of *SmGRAS 1~5* in *S*. *miltiorrhiza*. (DOCX)
