# Supplementary material for: Molecular cloning and characterization of five SmGRAS genes associated with tanshinone biosynthesis in Salvia miltiorrhiza hairy roots
Source: PLoS One. 2017 Sep 27;12(9):e0185322. doi: 10.1371/journal.pone.0185322 (PMC5617194; doi:10.1371/journal.pone.0185322)
Supplement: S1 Table — (DOCX) [file pone.0185322.s001.docx]

**S1 Table The clone primers of *SmGRAS1~5***

| **Gene ID** | **Forward primer** | **Reverse primer** |
| --- | --- | --- |
| ***SmGRAS1*(KY435886)** | GTGGGTCATTTGATTAGATGGATAC | CTACTACACACAGCGGAGTTC |
| ***SmGRAS2*(KY435887)** | TAACATATGGAATCTCATTGC | GTACTAATGCCAAGCAGAAG |
| ***SmGRAS3*(KY435888)** | CGTTCTCATGGATCCTTGGT | ATCAGACTCTATGCAGGGAC |
| ***SmGRAS4*(KY435889)** | TTGAGATGCAGGCATCCCAG | TCTGCGTTTATCCCCAAGCAG |
| ***SmGRAS5*(KY435890)** | GAGATGAGTTCTGCTACTGG | AAGGTCAGTGTTTGGGAAGC |
