# Supplementary material for: Molecular cloning and characterization of five SmGRAS genes associated with tanshinone biosynthesis in Salvia miltiorrhiza hairy roots
Source: PLoS One. 2017 Sep 27;12(9):e0185322. doi: 10.1371/journal.pone.0185322 (PMC5617194; doi:10.1371/journal.pone.0185322)
Supplement: S2 Table — (DOCX) [file pone.0185322.s002.docx]

**S2 Table The qRT-PCR primers of *SmGRAS* 1~5**

| **Gene ID** | **Forward primer** | **Reverse primer** |
| --- | --- | --- |
| ***SmGRAS1*(KY435886)** | GCCTACGACCAATCCTCCTACTCCA | CGGTCATGCGGCTGAACAATGC |
| ***SmGRAS2*(KY435887)** | ATCACCACATACGCCACGCTCTC | GCCACCAGCTTGTCGAATTCATCC |
| ***SmGRAS3*(KY435888)** | CCTTGCTCTACGTGCTGCTGAGAA | ACTGCTGCTTGTATACTCGCTGGA |
| ***SmGRAS4*(KY435889)** | AAGCCAGGGTGCCGAACTTTCTTT | CATTCCTCAACATCCACAGCGATTGC |
| ***SmGRAS5*(KY435890)** | TGGAGGAGACGGGTAGGCAACT | ACCATCGTCACCAGACTGTAGGC |
| ***SmCPS1*** | CCACATCGCCTTCAGGGAAGAAAT | TTTATGCTCGATTTCGCTGCGATCT |
| ***SmKSL1*** | TGGAAACAGTGTGACCCTTCTGCT | GCTTGCATACAAATAACACCCAATCCT |
| ***SmActin*** | GGTGCCCTGAGGTCCTGTT | AGGAACCACCGATCCAGACA |
