# Supplementary material for: Molecular cloning and characterization of five SmGRAS genes associated with tanshinone biosynthesis in Salvia miltiorrhiza hairy roots
Source: PLoS One. 2017 Sep 27;12(9):e0185322. doi: 10.1371/journal.pone.0185322 (PMC5617194; doi:10.1371/journal.pone.0185322)
Supplement: S3 Table — (DOCX) [file pone.0185322.s003.docx]

S3 Table Sequence features analysis of *SmGRAS 1~5* in *S. miltiorrhiza*

| **Name** | **Gene ID code** | **pI** | **Mw(Da)** | **NLS position** | | **AA Len** | **Group** **(subfamilies)** |  | |
| --- | --- | --- | --- | --- | --- | --- | --- | --- | --- |
| ***SmGRAS1*** | **KY435886** | **5.66** | **54680.04** | | **426-450** | **489** | **III(SHR)** |  |  |
| ***SmGRAS2*** | **KY435887** | **5.92** | **51214.97** | | **61-91** | **459** | **I(SCL)** |  |  |
| ***SmGRAS3*** | **KY435888** | **5.04** | **83937.26** | | **287-321** | **748** | **II(SCL)** |  |  |
| ***SmGRAS4*** | **KY435889** | **5.38** | **58142.56** | | **168-246** | **526** | **I(SCL)** |  |  |
| ***SmGRAS5*** | **KY435890** | **5.63** | **37542.17** | | **29-87** | **335** | **VIII(SCL)** |  |  |
